# Experimental and Numerical Study of Crater Volume in Wire Electrical Discharge Machining

**DOI:** 10.3390/ma13030577

**Published:** 2020-01-26

**Authors:** Jun Wang, José. A. Sánchez, Borja Izquierdo, Izaro Ayesta

**Affiliations:** 1 Aeronautics Advanced Manufacturing Center, CFAA (UPV/EHU), Bizkaia Technology Park, Building 202, 48170 Zamudio, Spain; joseantonio.sanchez@ehu.eus (J.A.S.); izaro.ayesta@ehu.eus (I.A.); 2 Department of Mechanical Engineering, University of the Basque Country, Plaza Torres Quevedo 1, 48013 Bilbao, Spain; borja.izquierdo@ehu.eus

**Keywords:** WEDM, thermal model, single discharge

## Abstract

Wire Electrical Discharge Machining (WEDM) is a popular non-conventional machining technology widely used in high-added value sectors such as aerospace, biomedicine, and the automotive industry. Even though the technology is now ready to meet the requirements of the most complex components, certain fundamental aspects related to the discharge process and gap conditions are not yet fully explained and understood. Combining single discharge experiments with numerical simulation represents a good approach for obtaining a deeper insight into the fundamentals of the process. In this paper, a fundamental study of the WEDM through single discharge experiments and numerical simulation is presented. WEDM single discharge experiments are described with the aim of identifying the relation between crater dimensions, discharge gap, and part surface roughness. A thermal transient numerical model of the WEDM process is presented, and correlation with actual industrial material removal rates (MRR) is analyzed. Results from single discharge WEDM experiments show that crater volume is as much as 40% lower when discharging on a rough surface than when the discharge occurs on a flat surface. The proposed thermal numerical model can predict actual removal rates of industrial machines with great accuracy for roughing cuts, deviations with experimental values being below 10%. However, lager deviations have been observed for other WEDM conditions, namely trim cuts, thus confirming the need for future research in this direction.

## 1. Introduction

Wire Electrical Discharge Machining (WEDM) is a non-conventional machining method for the manufacturing of high added-value components for aerospace, tooling and biomedical implants. In these areas, some super alloys are always adopted to be the best choice of material such as Nimonic C 263 [1], Inconel 718 [2], and so on. WEDM is not only capable of overcoming difficulties in super alloy machining, but also can meet the precise dimensional tolerance. A recent and comprehensive state of the art on the current situation of the EDM process (including WEDM) for processing stainless steels can be found in the work of Qudeiri [3]. Due to the large number of variables involved, process optimization is carried out in many cases following empirical approaches. A good and interesting example can be found in the work of Ishfaq [4], in which, by using Taguchi-based grey relational analysis, simultaneous optimization of process objectives such as cutting speed, surface roughness, and kerf width is successfully performed for the WEDM process of stainless steel. Due to the thermal nature of the process, metallurgical considerations for the machined surface are also a hot topic in research about the EDM and WEDM processes [5,6]. Following trends from data analytics, recent works propose gaining a deeper understanding about the WEDM by using artificial intelligence techniques, including unsupervised learning [7] and deep learning [8] approaches.

Theoretical models are a solid base for understanding the behavior of machining processes. Large research efforts are placed on the EDM processes in order to generalize conclusions from experimental works. In this context, the combination of simulative experiments and numerical simulation becomes a realistic tool for acquiring scientific knowledge. For the EDM processes, thermal simulation is probably the most used modelling tool [9]. From the literature, it can be observed that existing heat transfer models cannot successfully simulate actual removal rates when comparing with single discharge experiments, as shown in the work of Klocke [10] for the SEDM process. Thermal models are commonly applied to SEDM. For instance, in the Izquierdo paper [11], a multiple discharge model for the SEDM is presented, showing very good agreement between experiments and simulations in terms of material removal rate, surface roughness, heat affected zone, and recast layer.

As said before, the study of single discharge experiments can be found in literature as a tool for fundamental research in SEDM. One example of such fundamental research can be found in [12]. Satisfactory results were obtained about the environment and the distribution in which spark exists (liquid or gap), but also about nature and size of discharge crater. In these works, the influence of surface roughness and discharge gap on crater dimensions in SEDM was discussed. However, no information about these issues can be found particularly about the WEDM process. Probably, one of the most complete experimental works in scientific literature about fundamentals of SEDM is the one by Descouedres [13]. In this research, measurements about the nature, size and growth of the plasma channel in SEDM are described. Descouedres admits that his findings cannot be directly applied to WEDM because of the completely different boundary conditions. For instance, discharge duration is tens or even hundreds of μs in SEDM, on-time being in current WEDM machines about 2 μs. Other important differences are dielectric composition (especially referring to specific resistance of deionized water), flushing pressure within the gap, gap width, shape of current and voltage signals, etc.

From the literature review, it can be concluded that most of the research on thermal models and single discharge experiments correspond to the SEDM process. To the best knowledge of the authors, very little or no information is available about single discharge experiments in WEDM. In addition, thermal models of the WEDM process are scarce, and agreement with actual Material Removal Rates is limited. At this point, it must be mentioned that thermal models in WEDM have normally focused on studying the important problem of wire breakage [14], which limits the performance of WEDM machines, rather than on understanding process fundamentals. 

In this paper, a fundamental study of the WEDM through single discharge experiments and numerical simulation is presented. WEDM single discharge experiments are described in Section 2 with the aim of identifying the relation between crater dimensions, discharge gap, and part surface roughness. In Section 3, a thermal transient numerical model of the WEDM process is presented, and correlation with actual industrial Material Removal Rates is analyzed. Results from single discharge WEDM experiments show that crater volume is as much as 40% lower when discharging on a rough surface than when the discharge occurs on a flat surface. The proposed thermal numerical model can predict actual removal rates of industrial machines with great accuracy for roughing cuts, deviations with experimental values being below 10%. However, lager deviations have been observed for other WEDM conditions, namely trim cuts, thus confirming the need for future research in this direction.

## 2. Experimental Observations of Crater Dimensions in Single Discharge WEDM Experiments

### 2.1. Experimental Set-up

Because the removal capacity of the WEDM process is largely influenced by gap contamination and width, single discharge experiments can be used to clarify some of the aspects that cannot directly be obtained from the continuous WEDM process. As explained in the literature review, little or no information is available on single discharge WEDM experiments. A set of experiments involving single discharge WEDM’ing was carried out with the objective of achieving a deeper insight into crater geometry and removal capacity per discharge. The objective of the experiments was to obtain data on the influence of gap width, part surface roughness, and discharge current on crater diameter and depth (d_c_). The experiments are therefore run under conditions in which flushing is excellent and gap contamination is very low because the wire is not surrounded by the machined kerf.

Table 1 lists the WEDM settings used in the experiments. The part material was AISI D2 tool steel, and CuZn37 wire of 0.25 mm in diameter was used. These conditions correspond to the roughing cut (*h* = 50 mm) for the ONA AV35 WEDM machine where the experiments were conducted. Using the parameters of the first roughing cut, single discharge experiments were conducted with varying theoretical gap width (*g*) and part surface roughness, as described in Table 2. In all of the experiments, a minimum of 10 craters were machined. Three different parts with increasing surface roughness were prepared: a ground flat surface; a WEDM’ed surface using second trim cut parameters (these parameters are also shown in Table 1); and a WEDM’ed surface using the roughing cut parameters. The values of R_a_ and R_t_ for each surface are shown in Table 2. Two levels of theoretical gap width (as given by the position of wire guides) were used, which required a total of six experiments. 

**Figure 1:**
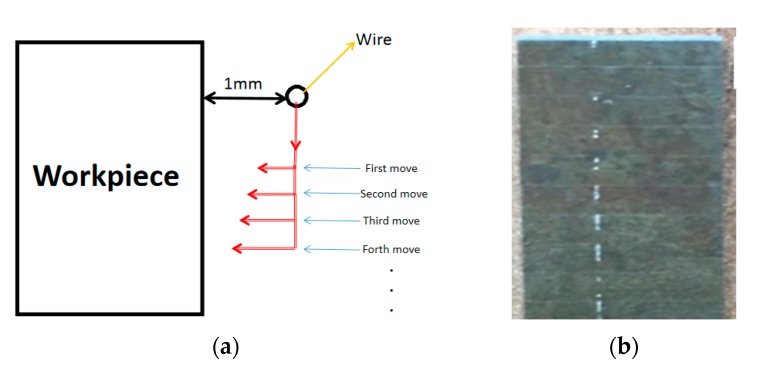
(**a**) Top view of the experimental principle; (**b**) single craters on part surface.

To produce individual craters, finding the critical point at which the first few sparks occur is essential. As shown in Figure 1a, firstly, the gap between wire and workpiece is controlled at 1 mm and then the wire is gradually positioned closer to the workpiece with every move until the first few sparks occur. It was found that the first spark occurred when the gap width was reduced to 20 μm. Figure 1b shows some of the individual craters. Crater geometry is then observed using the Leica DCM3D measuring microscope (Leica, Wetzlar, Germany).

### 2.2. Influence of Part Surface Roughness

Table 3 lists the results of single discharge crater measurement for the different values of part surface roughness (as given by R_t_). In addition, based on the pilot experiment, which is described in the previous section, it is known that the first spark will occur at a theoretical gap width of 20 μm; therefore, 20 μm and 10 μm were the values considered in this experiment. In terms of crater diameter (*D_c_*), depth (*d_c_*) and volume (*V_c_*) (assuming spherical cap crater), average values, and standard deviations are also presented in Table 3.

Initial inspection of the results reveals a notable influence of part surface roughness on crater dimensions. A flat surface on which the presence of peaks is scarce produces larger crater diameters. A similar trend is observed in the experiments with a theoretical gap width 10 μm and 20 μm. In both cases, the average crater diameter is greater than 100 μm. 

For the same gap width, an increase in R_t_ from 3.59 μm to 5.92 μm leads to a reduction in crater depth (d_c_), and therefore a considerable reduction in crater volume of approximately 35%, regardless of the gap width. In this case, there is no variation in crater diameter. However, when further increasing the peak height up to 15.27 μm, crater diameter is reduced by approximately 18% (97.5 μm in the case of 10 μm gap width, and 87 μm in the case of a 20 μm gap). This trend is observed both with gap widths of 10 μm and 20 μm. As a consequence, when comparing the single discharge on the flat surface with that on a WEDM’ed surface obtained using roughing cut conditions, crater volume is reduced by as much as 40%. 

**Figure 2:**
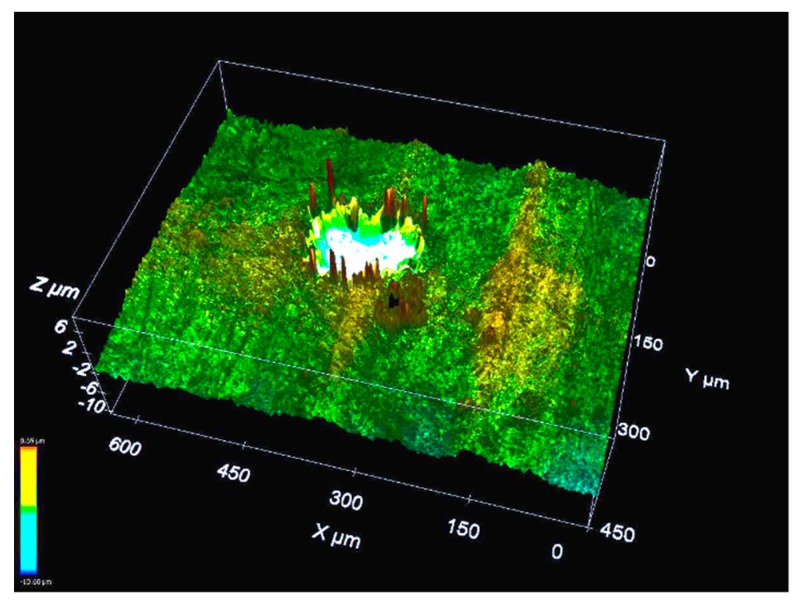
Rim generated by a single discharge on a ground surface (R_a_ 0.46 μm, R_t_ 3.59 μm). Rim height can be as much as 8 μm.

**Figure 3:**
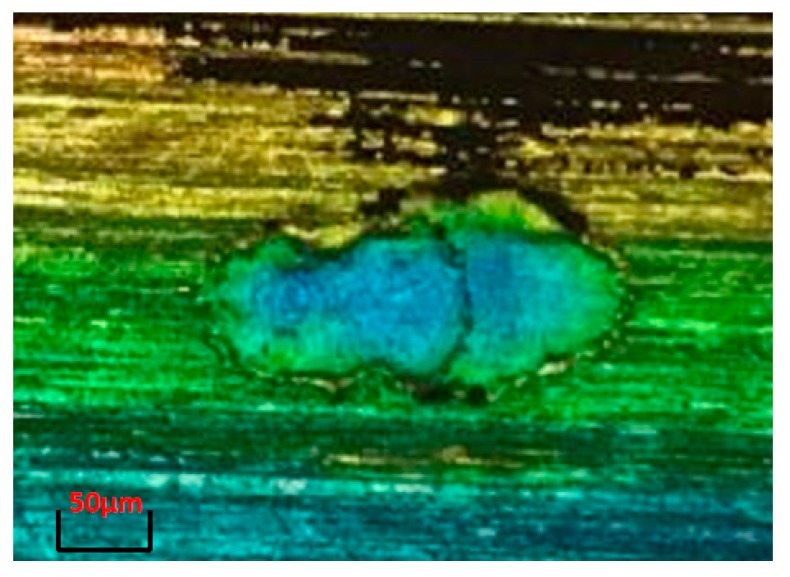
Occurrence of two consecutive single craters on a ground surface.

A further insight into the role of roughness peaks can be gained by examining the cause of the formation of consecutive craters. To do this, a single crater which is obtained under the same electrical parameters as those listed in Table 1 is scanned by a measuring technique called dual-core DCM 3D by Leica, which is a measuring microscope that combines confocal and interferometry technologies. This technique provides high-speed measurements with high resolution down to 0.1 nm. Therefore, this is a fast and non-destructive 3D measurement in which no sample preparation is required. The scanning result is shown in Figure 2. The experiment corresponds to a single discharge on the flat (ground) surface with a theoretical gap width of 10 μm. A rim of melted and non-removed material is clearly present on the periphery of the crater. Rim height can be as much as 8 μm through observation of Figure 2, which is a value similar to the measured crater depth. The morphology of two consecutive single craters when occurring on a flat surface, which is shown in Figure 3, is also obtained through Leica DCM3D. Unfortunately, the focus limit of the measuring instrument does not allow a better quality for Figure 3. However, the shape of the consecutive craters can be clearly observed and this contributes to the better understanding of the results. The diameters of the craters in this example are 127 μm (first crater) and 114 μm (second crater), respectively. Consecutive craters are, in all cases, smaller in diameter than the first single crater on the flat surface.

**Figure 4:**
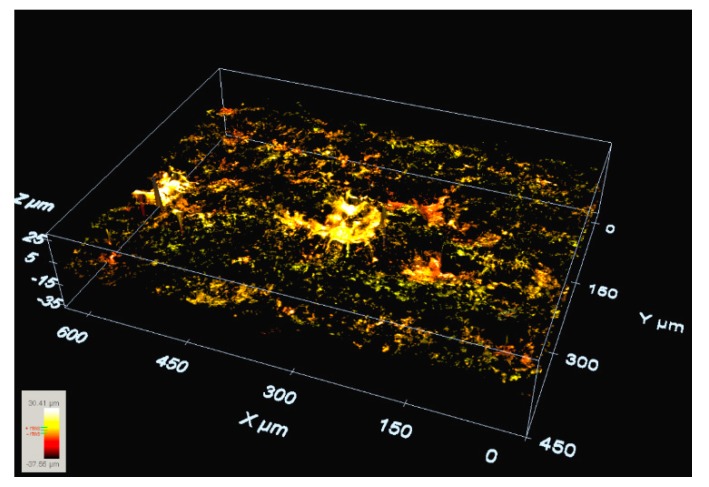
Single crater on a WEDM’ed surface (roughing cut, R_a_ 2.60 μm, R_t_ 15.27 μm). The effect of the rim is clearly less pronounced than in the case of Figure 2.

Figure 4 shows the crater scanning resulting by Leica DCM3D that is generated by a single discharge occurring on a surface WEDM’ed in roughing cut conditions. The occurrence of consecutive discharges has been measured on both flat and rough surfaces. In the case of a flat surface, in 47% of the cases, consecutive discharges were observed, concentrated on the rim of a previous crater. In the case of the rough surface (WEDM roughing cut), this percentage falls to 18%. Therefore, the probability of discharge concentration is clearly lower when discharge occurs on the rough surface. Through the 3D scanning of a single crater on the rough surface, the rim of the crater is less apparent than that observed on the flat surface. As stated above, the rim of a previous crater primarily affects single discharge location in WEDM on a flat surface, but this effect becomes less marked on a rough surface due to the presence of multiple peaks and valleys on the surface. 

### 2.3. Influence of Gap Width

From the results displayed in Table 3, it can be deduced that, firstly, an increase from 10 μm to 20 μm does not produce a significant variation in crater dimensions. Secondly, surface roughness imposes stronger boundary conditions on heat partition than those imposed by gap width. However, it is known that a larger gap width results in a greater plasma channel diameter, and thus reduced heat flux to the electrodes. Furthermore, a longer plasma channel can increase energy losses towards dielectric, thus reducing heat conduction towards the workpiece and wire electrodes. 

As explained previously, the above simulative experiments occurred under conditions in which flushing is excellent and gap contamination is very low because the wire is not surrounded by the machined kerf. This is the reason why very small values of theoretical gap width can be set (10 μm and 20 μm). These values differ considerably from the real-life values of WEDM industrial practice. Therefore, in this section, analysis of the influence of gap width will be addressed. Since during actual practice discharges occur on a surface where WEDM craters have already been generated, comparison will be carried out using the experiments corresponding to WEDM roughing cut (that is, initial surface roughness R_a_ 2.60 μm, R_t_ 15.27 μm). Using the same electrical parameters as those listed in Table 1, different values of gap width can be achieved by cutting different part thicknesses (h). Table 4 lists the values of part thickness and the corresponding values of gap width (as given by machine tables) during the new set of experiments.

Because the WEDM process is stochastic in nature, results from single discharge experiments were compared with the average removal capacity per discharge during WEDM under industrial conditions. This can be obtained by using Equation (1):

(1) $$V_{discharge}=\frac{h\cdot w\cdot l}{N_{discharge}}$$

where *V_discharge_* (mm^3^/discharge) is the average removal capacity per discharge, *h* is part thickness (mm), *w* is kerf width (mm), *l* is the cutting length (mm), and *N_discharge_* is the total number of discharges. This number can be directly measured using a built-in application in the machine generator. 

**Figure 5:**
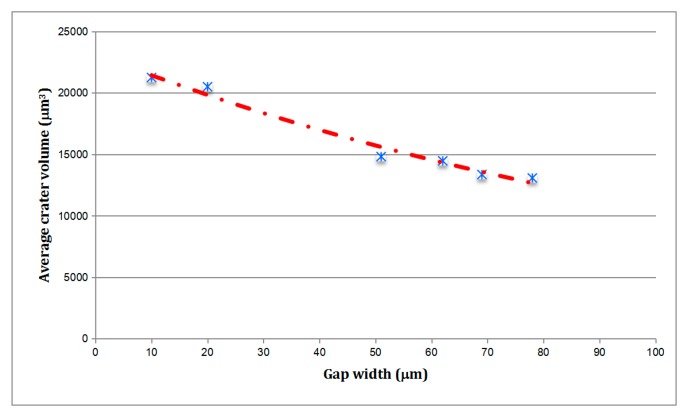
Average removal capacity per discharge vs. gap width (discharge surface roughness corresponding to roughing cut, R_a_ 2.60 μm, R_t_ 15.27 μm).

Figure 5 shows the evolution of average removal capacity per discharge (mm^3^) as a function of gap width. The above results quantify for reduction of crater volume (average removal capacity per discharge) associated with the increase in gap width. Within the range of industrial WEDM conditions (gap width between 50 μm and 80 μm), the differences are less marked, the reduction in average crater volume between these conditions being no higher than 11%. However, when comparing crater volume for s gap width of 10 μm (single discharge experiments) and for a gap width of 80 μm (continuous WEDM, *h* = 250 mm), the reduction in average crater volume becomes as high as 36%. It is important to consider that surface roughness and electrical settings are similar in this set of experiments, and thus the variation can only be attributed to differences in gap width.

Upon inspection of the results, it can be concluded that both gap width and part surface roughness impose strong boundary conditions on heat partition that must be taken into account in any fundamental study of the process. 

## 3. Numerical Simulation of the WEDM Industrial Process

**Figure 6:**
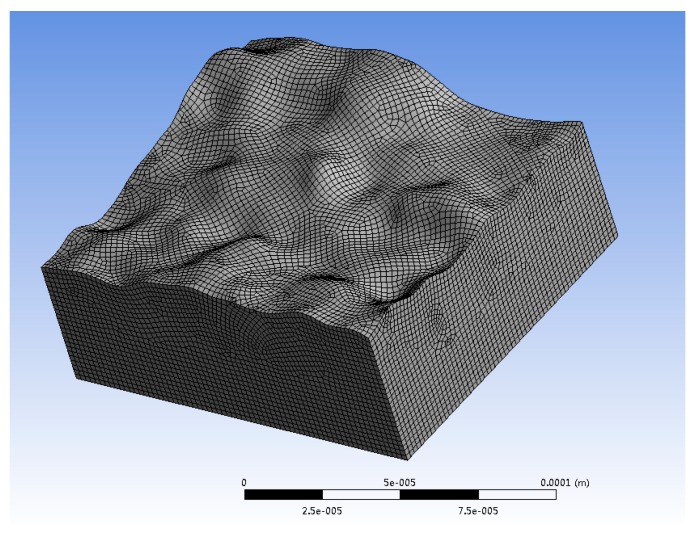
Meshing of the WEDM’ed rough surface for solving the thermal model.

In order to better understand heat flux conditions into the workpiece, a transient thermal model for single discharge in WEDM was developed using the finite element method (FEM) commercial software ANSYS (Version 19.1, ANSYS, Canonsburg, PA, USA). The actual rough surface (as WEDM’ed using conditions of roughing cut) was imported, meshed by Hex dominant method and element size 2 μm (Figure 6), and the thermal transient problem was solved on this surface in order to quantify the effect of part geometry on heat transfer.

The classical Fourier heat transmission Equation (2) is used to model the heat transfer problem, where *T* is temperature, *t* is time, *ρ* is the density, *C_p_* is the specific heat, and *K* is the thermal conductivity. Variables *r* and *z* are the distances to the heat center along the horizontal direction and vertical direction, respectively [11]:

(2) $$\frac{1}{r}\frac{\partial }{\partial r}\left(K_{t}r\frac{\partial T}{\partial r}\right)+\frac{\partial }{\partial z}\left(K_{t}\frac{\partial T}{\partial z}\right)=\rho C_{P}\frac{\partial T}{\partial t}$$

The heat flux can be seen as only applying on the top of the spark contact surface. The rest of surface is in a convection that is applied to release energy towards the dielectric (deionized water) [15]. In EDM, a Gaussian heat source is used as described in [16], and expressed by Equation (3):

(3) $$Q\left(r\right)=\frac{4.57f_{c}UI}{\pi R_{P}^{2}}\exp\left(-4.5\frac{r^{2}}{R_{P}^{2}}\right)$$

Actual voltage and current signals were measured using a Tektronix 5034B high frequency Digital Oscilloscope, a Tektronix ThDP0200 Differential Voltage probe and a PEM CWT1xR Current probe (Tektronix, Beaverton, OR, USA). The model is fed with these values (*U* measured voltage, *I* measured current). The variable *f_c_* is the fraction of heat to the workpiece. A value of 40% has been taken from reference [17]. *R_p_* is the radius of the plasma channel. In this work, it was decided to use the model described in [18] that predicts a value of *R_p_* of 81 μm after 2 μs, as given by Equation (4):

(4) $$R_{p}=40.5\cdot t$$

Latent heats of melting and evaporation were also considered in the model. For the properties of the AISI D2 steel used in the experiments, the values listed in Table 5 were provided by the manufacturer of the steel.

The first objective was to study if surface roughness imposes any type of difference on heat conduction towards the workpiece. Since the flushing conditions during single discharge experiments are excellent, water was chosen as the convection environment in the simulation of a single crater. Furthermore, in single discharge experiments, flushing efficiency is very high, and, therefore, only for a first approach, it was assumed that all the material above the melting temperature is removed from the workpiece (in other words, flushing efficiency is 100%). Of course, this is not true, and corrections will be applied later on.

**Figure 7:**
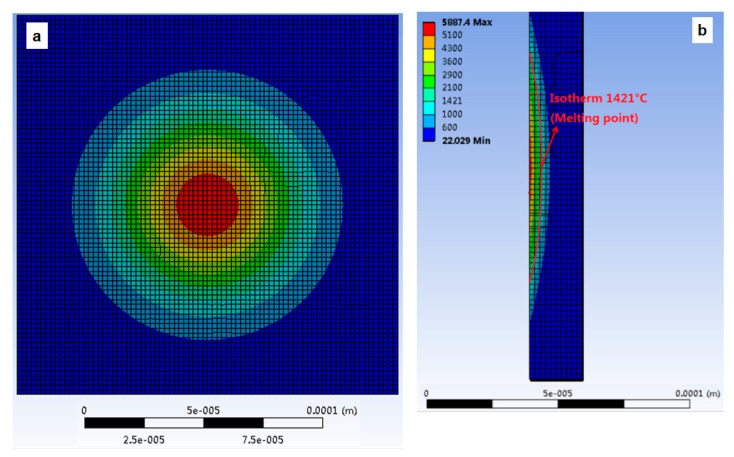
Numerical simulation of crater volume. Single discharge on a flat surface: (**a**) Front view (**b**) lateral view.

**Figure 8:**
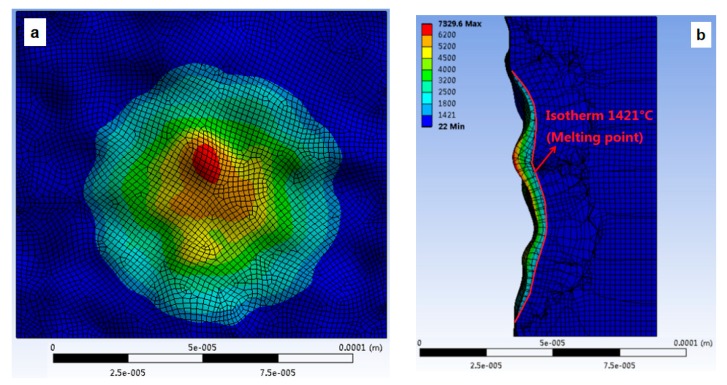
Numerical simulation of crater volume. Single discharge on a rough surface, as produced by WEDM roughing cut: (**a**) Front view (**b**) lateral view.

Figure 7 and Figure 8 show the geometry of the volume of material removed in the case of simulation with the heat source applied on the flat and on the rough surfaces under the same heat flux conditions (similar *f_c_* and *R_p_*). ANSYS APDL (Version 19.1, ANSYS, Canonsburg, PA, USA) is used to calculate the volume of part material removed by the discharge. For the simulation on the flat surface, crater volume is 16223.73 μm^3^. In the case of rough surface, the simulation volume of material removed is 17,216.11 μm^3^. As a result, the difference is 5.76%.

**Figure 9:**
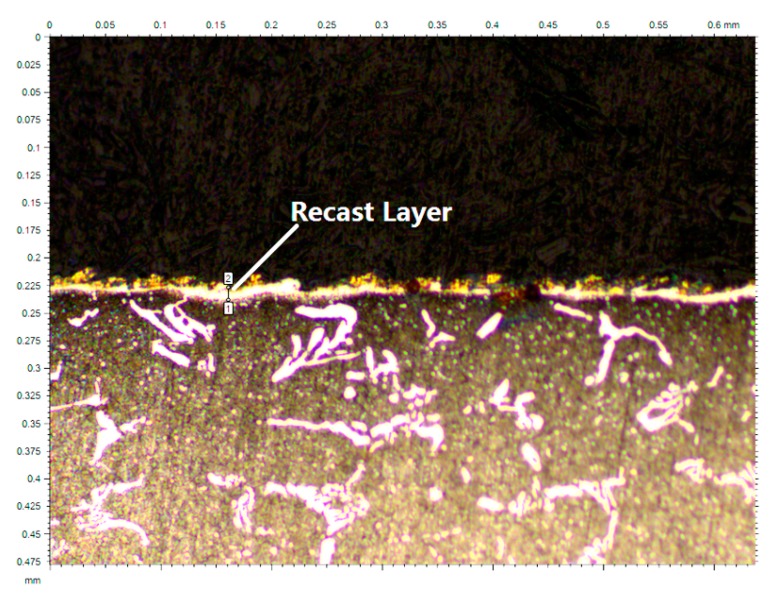
Recast layer after WEDM under the conditions described in Table 1.

Clearly, flushing efficiency in actual WEDM is not 100% and must be considered. The thickness of the recast layer can be used to estimate flushing efficiency. Recast layer accounts for the material that has undergone over melting temperature, but it has not been actually removed. The result is a brittle martensitic layer that remains on the machined surface. Figure 9 shows a micrograph gotten from Leica DCM3D. It displays clearly a WEDM’ed surface (after and metallurgical preparation with Remet IPA 40 (in conditions of 200 °C and 6 bar) and polishing with Reset LS1) machined under the conditions described in Table 1. The average thickness of the recast layer for the experiment is 12.4 μm. Using the total number of discharges, an average volume of recast material per discharge can be calculated. The resulting value is 911.63 μm^3^, a value of flushing efficiency of 93%–94%.

Now, the accuracy of the numerical model can be analyzed. Considering flushing efficiency, the volume of a single crater obtained from numerical simulation is 15312.1 μm^3^. From the results in Table 3 and Figure 5, the window of optimum application of the numerical model can be established. In fact, deviations between numerical and experimental results increase with a decrease in gap width and a reduction of the roughness of part surface. As an example, the volume of the simulated crater is only 43% of the actual volume of the crater from the experiment (35,648.49 μm^3^) generated with gap width 10 μm and part surface finish corresponding to ground state R_a_ 0.46 μm (single discharge experiment). In other words, the model does not simulate adequately the heat transfer problem for these WEDM conditions. Deviation is still unacceptable, but clearly smaller, in the case of the single discharge experiment with gap width 10 μm and part surface finish corresponding to WEDM roughing cut R_a_ 2.60 μm. For this case, the volume of the simulated crater is 72% of the volume of the crater from the experiment (21,255.18 μm^3^). 

However, when comparing numerical results with the actual removal capacity of the WEDM machine in conditions of roughing cut (Figure 5, gap width between 50 μm and 80 μm), it can be observed that the simulation method produces excellent results. Comparison is presented in Table 6.

Deviations between simulated and experimental values are, for industrial conditions, below 10% (see Table 6), with a minimum value of 2.7%. The model exhibits an excellent agreement with industrial practice for predicting crater volume (and therefore, Material Removal Rate) for WEDM roughing cuts. However, as explained above, agreement is very poor for the other set of conditions. As a conclusion, it can be stated that the values of the boundary conditions (mainly *f_c_* and *R_p_*) existing in literature apply only to the WEDM conditions corresponding to the roughing cut (first cut), but, since the heat transfer problem is largely affected by gap width and surface roughness, these values are not useful for simulation of trim cuts. Future work will focus on determining the laws that relate *f_c_* and *R_p_* with gap width and part roughness, thus producing a complete model of the WEDM process.

## 4. Conclusions

On the basis of the work conducted, the following conclusions can be drawn:

- WEDM single discharge experiments on an industrial machine show the dependency of crater dimensions on discharge gap and part surface roughness. The volume of part material removed per discharge can be as much as 40% lower when discharging on a WEDM’ed surface (roughing cut, R_a_ 2.60 μm, R_t_ 15.27 μm) than when discharging on a low surface roughness (as ground, R_a_ 0.46 μm, R_t_ 3.59 μm). The results indicate that, for a given gap width, crater volume increases significantly with a reduction of the roughness of the discharge surface.
- Single discharge experiments have generated results that are consistent with the known fact that smaller gap widths produce larger crater volumes. Thus, for a given part surface roughness, crater volume decreases from 21,255 μm^3^ to 20,506 μm^3^ when the discharge gap increases from 10 μm to 20 μm. Even though flushing conditions within the gap are very different, under similar electrical settings in industrial WEDM, the average volume removed per discharge further falls to 13080 μm^3^ with a gap value of 78 μm. It can further verify the influence of gap width on the removal capacity of discharge.
- A numerical model based on the resolution of the transient heat transfer problem from the discharge to the workpiece has been set up using the Finite Element Method software ANSYS. The heat conduction problem has been solved both on a flat and on a rough surface and, as a result, the difference is no more than 6%. This result shows that the contribution of roughness on crater volume is very limited.
- The degree of agreement between the numerical model and experimental results has been analyzed and the deviations are within the range 2.7–9.4%. Therefore, it can be said that the model exhibits an excellent agreement with industrial practice for predicting crater volume (and therefore, Material Removal Rate) for WEDM roughing cuts.
- However, the degree of agreement is largely affected by gap width and initial part surface roughness. As an example, the volume of the simulated crater is only 43% of the actual volume of the crater from the experiment (35648.49 μm^3^) generated with gap width 10 μm and part surface finish corresponding to ground state R_a_ 0.46 μm (single discharge experiment). In other words, the model does not simulate adequately the heat transfer problem for other WEDM conditions different from the first roughing cut.
- Since the heat transfer problem is largely affected by gap width and surface roughness, these values are not useful for simulation of trim cuts. Future work will focus on determining the laws that relate *f_c_* and *R_p_* with gap width and part roughness, thus producing a complete model of the WEDM process.

## Figures and Tables

**Table 1:** Electrical parameters for single discharge experiments.

WEDM Parameters	Roughing Cut	1st Trim Cut	2nd Trim Cut
*I* (A)	5	5	3
*U_0_* (V)	80	100	90
*U_s_* (V)	52	48	10
*f* (mm/min)	12	10	10
*t_on_* (μs)	2	2	2
*t_off_* (μs)	12	9	1
*h* (mm)	50		
Dielectric	Deionised water		

**Table 2:** Gap width and initial part surface roughness for the experiments.

*g* (μm)	Flat (ground) Surface	2nd Trim Cut	Roughing Cut
10 μm; 20 μm	R_a_ (μm): 0.46	R_a_ (μm): 0.81	R_a_ (μm): 2.60
	R_t_ (μm): 3.59	R_t_ (μm): 5.92	R_t_ (μm): 15.27

**Table 3:** Crater dimensions vs. part initial roughness and theoretical gap width.

*G* (μm)	*R_t_* (μm)	*D_c_* (mm)		*d_c_* (μm)		*V_c_* (μm^3^)
		Average	STD	Average	STD	Average
10	3.59	106	16.2	7.7	1.3	35,648.49
10	5.92	105.7	27.7	4.8	0.7	23,168.42
10	15.27	97.5	21.6	5.1	1.2	21,255.18
20	3.59	104	21	6.8	0.9	34,725.21
20	5.92	102	21.8	5.4	0.9	23,603.25
20	15.27	87	16.1	6.3	1.6	20,505.93

**Table 4:** Theoretical gap width values in the experiments.

*H* (mm)	*g* (μm)
NA (Single discharge, rough surface)	10
NA (Single discharge, rough surface)	20
20	51
50	62
150	69
250	78

**Table 5:** Thermal properties of AISI D2 tool steel.

*T* (K)	*T_m_* (K)	*ρ* (kg/m^3^)	*λ* (W/(m·°C))	*C* (J/(kg·°C)
293.15	1658.15	7700	20	460
473.15		7650	21	
673.15		7600	23	

**Table 6:** Comparison between experimental and simulation results.

	*V_discharge_* (μm^3^)	*Error* (%)
Industrial WEDM values (after Figure 5, gap 50–80 μm)	13991–15731	
Numerical results (including plasma flushing efficiency)	15312.1	2.7–9.4

## Abbreviations

The following abbreviations are used in this manuscript:

*C*	Specific heat (J/(kg·°C))
*D_c_*	Crater diameter (mm)
*d_c_*	Crater depth (µm)
*Error*	Deviation between simulated value and experimental value (%)
*f*	Wire infeed rate (mm/min)
*f_c_*	Fraction of total heat transferred to workpiece (%)
*FEM*	Finite element method
*g*	Theoretical gap width (µm)
*h*	Part thickness (mm)
*I*	Current (A)
*l*	Cutting length (mm)
*MMR*	Material removal rates
*N_discharge_*	Total number of discharges
*Q(r)*	Gaussian heat flux
*r*	Distance to the center of heat source (µm)
*R_a_*	Roughness Average (µm)
*R_p_*	Plasma channel radius (µm)
*R_t_*	Total height of the roughness profile (µm)
*SEDM*	Sinking Electrical Discharge Machining
*STD*	Standard deviation
*T*	Temperature (K)
*t*	Time (µs)
*T_m_*	Melting temperature (K)
*U*	Voltage (V)
*U_s_*	Servo voltage (V)
*V_c_*	Crater volume (µm^3^)
*V_discharge_*	Average removal capacity of each discharge (mm^3^/discharge)
*w*	Kerf width (mm)
*WEDM*	Wire Electrical Discharge Machining
*ρ*	Density (kg/m^3^)
*K*	Thermal conductivity (W/(m·°C))
